# Asthma control perception by caregivers: Quality of life and spirometric findings among children

**DOI:** 10.6026/973206300221949

**Published:** 2026-04-30

**Authors:** Adithi Umesh Basappa, Madhavi S Walulkar

**Affiliations:** 1 Department of Emergency Medicine, ETCM Hospital, Karnataka, India; 2 Department of Physiotherapy, NKP Salve Institute of Medical Sciences and Research center and Lata Mangeshkar Hospital, Nagapur, India

**Keywords:** Asthma control, caregiver perception, quality of life, children, spirometry, pediatric asthma, asthma management, questionnaire-based study, lung function, caregiver awareness

## Abstract

Childhood asthma remains a major chronic respiratory disorder and misperception of disease control by caregivers contributes to suboptimal management and impaired quality of life. Therefore, it is of interest to assess caregiver perception of asthma control and examined its association with spirometric findings and quality-of-life scores among children aged 6-14 years. Caregivers completed a structured questionnaire and children underwent spirometry to measure FEV_1_, FVC and FEV_1_/FVC ratio according to standard guidelines. Although 46% of caregivers perceived asthma as well controlled, 56% of children demonstrated obstructive patterns on spirometry, with a significant association between perception and lung function (χ^2^=11.63, p=0.003). Spirometry showed strong positive correlations with quality-of-life scores (r=0.67, p<0.01), indicating that objective assessment combined with caregiver education is essential for optimal pediatric asthma management.

## Background:

Asthma is one of the most common chronic respiratory diseases in children and remains a significant cause of morbidity worldwide [1]. Despite advances in pharmacotherapy and guideline-based management, a substantial proportion of children continue to have poorly controlled asthma [2]. Inadequate symptom recognition and suboptimal adherence to controller therapy contribute to persistent airway inflammation and functional limitation [3]. Caregivers play a central role in monitoring symptoms, administering medication and seeking medical care for children with asthma [4]. However, caregiver perception of asthma control often differs from objective clinical assessment, leading to overestimation or underestimation of disease severity [1]. Recent studies have demonstrated that discrepancies between subjective assessment and spirometric findings may delay therapeutic escalation and increase the risk of exacerbations [6, 7]. Spirometry remains the gold standard for objective evaluation of airflow limitation and is recommended for routine monitoring in pediatric asthma [8]. Beyond physiological impairment, asthma significantly affects physical functioning, emotional well-being and school performance in children [9]. Quality-of-life assessment provides additional insight into the psychosocial burden of uncontrolled asthma and complements objective lung function testing [10]. Therefore, it is of interest to evaluate caregiver perception of asthma control and analyse its association with spirometric findings and quality-of-life measures in children with bronchial asthma.

## Materials and Methods:

This cross-sectional observational study was conducted in the outpatient department of a tertiary care hospital. The study included 100 children aged 6-14 years with physician-diagnosed bronchial asthma and their primary caregivers. Participants were enrolled after obtaining written informed consent. Children with other chronic respiratory disorders or significant comorbidities were excluded. Caregiver perceptions of asthma control and child quality of life were assessed using a pre-validated structured questionnaire. The perception component evaluated symptom frequency, activity limitation and medication use. Quality of life was measured using a standardized pediatric asthma quality-of- life scale covering physical, emotional, school and social domains. Higher scores indicated better perceived quality of life. Spirometry was performed for all children according to standardized guidelines. Forced expiratory volume in one second (FEV_1_), forced vital capacity (FVC) and FEV_1_/FVC ratio were recorded. Lung function patterns were classified as normal or obstructive based on established reference criteria. Asthma control status was interpreted in accordance with contemporary guideline recommendations. Data were entered into a spreadsheet and analyzed using statistical software. Descriptive statistics summarized demographic and clinical characteristics. The association between caregiver perception and spirometry findings was assessed using the chi-square test. Correlation between perception scores, spirometric parameters and quality-of-life scores was evaluated using Pearson's correlation coefficient. Statistical significance was set at p < 0.05. Ethical approval was obtained from the Institutional Ethics Committee prior to study initiation. Confidentiality and anonymity of participants were maintained throughout the study.

## Results:

A total of 100 children with bronchial asthma and their caregivers were included in the analysis. The mean age of the children was 10.2 ± 2.4 years, with the majority aged between 9 and 11 years. There was a male predominance (58%) and most participants resided in urban areas (64%). Regarding clinical profile, 42% had disease duration of 1-3 years and 40% had asthma for more than three years. More than half of the children (55%) experienced one to two exacerbations in the previous year, while 31% reported three or more episodes. Prior hospitalization due to asthma was documented in 29% of cases. Most caregivers had at least secondary-level education (38%) and 58% belonged to the middle socioeconomic class. Based on caregiver perception, 46% of children were considered well controlled, 37% partly controlled and 17% poorly controlled. In contrast, spirometric evaluation revealed normal lung function in only 44% of children, while 56% demonstrated an obstructive pattern. Cross-tabulation showed that 39.1% of children perceived as well controlled had objective airway obstruction. A statistically significant association was observed between caregiver perception and spirometry findings (χ^2^ = 11.63, p = 0.003). The overall mean quality-of-life (QoL) score was 69.7 ± 10.5, with the lowest domain score observed in school performance (63.2 ± 11.5). Children classified as well controlled demonstrated significantly higher QoL scores (77.3 ± 8.6) compared to partly controlled (68.5 ± 9.8) and poorly controlled groups (59.2 ± 10.4) (p < 0.001). Medication adherence was reported as regular by 61% of caregivers, while 39% admitted inconsistent adherence. Significant positive correlations were observed between spirometry and QoL scores (r = 0.67, p < 0.01), caregiver perception and spirometry (r = 0.62, p < 0.01) and caregiver perception and QoL (r = 0.58, p < 0.01). Overall, the findings indicate a measurable discrepancy between subjective caregiver assessment and objective lung function, with significant implications for quality-of-life outcomes. Table 1 (see PDF) shows that the highest proportion of children (37%) was aged 9-11 years, with males accounting for 58% and urban residents constituting 64% of the sample. Table 2 (see PDF) indicates that 42% had asthma for 1-3 years, 55% experienced one to two exacerbations in the past year and 29% required prior hospitalization. Table 3 (see PDF) demonstrates that 38% of caregivers had secondary education and 58% belonged to the middle socioeconomic class. Table 4 (see PDF) reveals that 46% of caregiver's perceived asthma as well controlled, whereas 17% considered it poorly controlled. Table 5 (see PDF) indicates that 56% of children exhibited an obstructive spirometry pattern compared with 44% who had normal lung function. Table 6 (see PDF) demonstrates that 39.1% of children perceived as well controlled had objective airway obstruction, with a statistically significant association between perception and spirometry findings (χ^2^=11.63, p=0.003). Table 7 (see PDF) shows that the lowest quality-of-life domain score was school performance (63.2 ± 11.5), while social interaction had the highest mean score (75.8 ± 9.6). Table 8 (see PDF) indicates that mean QoL scores declined progressively from well controlled (77.3 ± 8.6) to poorly controlled asthma (59.2 ± 10.4), with statistical significance (p<0.001). Table 9 (see PDF) reveals that 61% of caregivers reported regular medication adherence, whereas 39% reported inconsistent adherence. Table 10 (see PDF) demonstrates strong positive correlations between spirometry and QoL (r=0.67), caregiver perception and spirometry (r=0.62) and moderate correlation between perception and QoL (r=0.58), all statistically significant (p<0.01).

## Discussion:

This study evaluated caregiver perception of asthma control and examined its association with spirometric findings and quality-of-life outcomes in children. The findings demonstrate a clear discrepancy between subjective caregiver assessment and objective lung function, with caregivers frequently overestimating asthma control [11]. Similar patterns have been reported in recent pediatric asthma studies, indicating that reliance on symptom-based perception alone may mask ongoing airway obstruction. This mismatch is clinically relevant, as delayed recognition of poor control may result in inadequate treatment escalation and increased risk of exacerbations [12]. Although nearly half of the caregivers perceived asthma to be well controlled, spirometry revealed obstructive patterns in more than half of the children. Objective lung function testing remains essential for accurate assessment of asthma control, particularly in children who may adapt to symptoms or underreport limitations. Caregiver perception is often influenced by episodic symptom relief or reduced use of rescue medication, which may not reflect underlying airway inflammation. These findings reinforce the limitations of subjective assessment when used in isolation [13]. The significant association between caregiver perception and spirometric findings indicates that perception is not entirely inaccurate but lacks sufficient sensitivity to detect subclinical disease. Comparable studies have shown that caregivers tend to recognize severe symptoms but may overlook persistent airflow limitation. This partial concordance suggests that caregiver education should focus on understanding the chronic and fluctuating nature of asthma rather than episodic symptom control alone. Integrating spirometry into routine follow-up can help bridge this perceptual gap [14]. Quality-of-life assessment provided additional insight into the functional impact of asthma control. Children with well-controlled asthma demonstrated significantly higher quality-of-life scores across physical, emotional and school domains. School performance emerged as the most affected domain, reflecting the broader psychosocial burden of uncontrolled asthma beyond respiratory symptoms. These findings align with contemporary evidence showing that asthma-related limitations significantly impair academic participation and daily functioning [15]. The strong positive correlation between spirometry and quality-of-life scores highlights the close relationship between physiological control and perceived well-being. Improved lung function was associated with better overall quality of life, emphasizing that objective disease control translates into meaningful functional benefits. The moderate correlation between caregiver perception and quality of life further suggests that caregiver views partially capture daily impact but may underestimate physiological severity [16]. Medication adherence emerged as an important contributing factor, with nearly two-fifths of caregivers reporting inconsistent use. Suboptimal adherence may explain, in part, the observed discordance between perceived control and spirometric obstruction. Previous studies have identified caregiver misunderstanding of maintenance therapy as a major barrier to sustained asthma control. Addressing adherence through targeted counseling may therefore improve both objective and subjective outcomes [17]. By integrating caregiver perception, spirometric assessment and quality-of-life evaluation, this study provides a multidimensional understanding of pediatric asthma control. The findings underscore that caregiver perception should not be the sole determinant of disease control. Routine spirometry combined with structured caregiver education and periodic quality-of-life assessment may lead to more accurate evaluation, timely intervention and improved long-term outcomes in children with asthma.

## Conclusion:

Caregiver perception of asthma control frequently differs from objective spirometric assessment, leading to potential under recognition of persistent airway obstruction. Objective lung function demonstrated strong associations with quality-of-life outcomes, underscoring the clinical relevance of routine spirometry in pediatric asthma management. Thus, integrating caregiver education, adherence monitoring and regular spirometric evaluation is essential to achieve accurate disease assessment and sustained improvement in child health and functional well-being.
